# Central Centrifugal Cicatricial Alopecia in Black Women: Diagnostic Challenges and Molecular Insights

**DOI:** 10.7759/cureus.116655

**Published:** 2026-09-21

**Authors:** Calista Persson, Alexis Coican, Sarah Ferrer-Bruker

**Affiliations:** 1 Osteopathic Medical School, Nova Southeastern University Dr. Kiran C. Patel College of Osteopathic Medicine, Fort Lauderdale, USA; 2 Department of Dermatology, HCA Florida Orange Park Hospital, Orange Park, USA

**Keywords:** black women, central centrifugal cicatricial alopecia, cicatricial alopecia, fibroproliferative pathways, hair follicle fibrosis, hair loss, histopathology, molecular mechanisms, scarring alopecia, trichoscopy

## Abstract

Central centrifugal cicatricial alopecia (CCCA) is a scarring alopecia that disproportionately affects Black women but remains underrecognized and frequently misdiagnosed because of its clinical overlap with other forms of hair loss. Emerging research has expanded the understanding of CCCA beyond its clinical presentation, highlighting the potential contribution of genetic susceptibility and disruptions in fibroinflammatory and metabolic pathways. A comprehensive literature review of publications from 2015 to 2025 identified 15 primary studies, supplemented by relevant reviews and case reports, addressing diagnostic features and evolving perspectives on disease pathogenesis. CCCA typically presents as progressive thinning of the central scalp and may resemble androgenetic alopecia or alopecia areata. Dermoscopy and histopathology remain important for accurate diagnosis, with findings that may include follicular dropout, perifollicular fibrosis, and lymphocytic inflammation. Molecular investigations have identified several pathways of interest, including those involving peptidyl arginine deiminase 3 (PADI3) and peroxisome proliferator-activated receptor gamma (PPAR-γ); however, their precise clinical significance continues to be investigated. Associations with autoimmune and metabolic comorbidities further support a multifactorial disease process involving genetic, inflammatory, and metabolic influences. Underrecognition may delay treatment and contribute to worse outcomes. Greater clinician awareness, culturally informed diagnostic approaches, and continued investigation of molecular mechanisms may support earlier detection, more individualized treatment, and reduced health disparities among women of color.

## Introduction and background

Central centrifugal cicatricial alopecia (CCCA) is a primary lymphocytic scarring alopecia that predominantly affects Black women, with onset commonly occurring between 30 and 55 years of age [1,2]. The disease typically begins with thinning at the vertex and progressively expands toward other regions of the scalp. Patients may experience pruritus, burning, tenderness, or dysesthesia, although hair loss may be the only presenting concern [3]. Persistent perifollicular inflammation and fibrosis can result in irreversible follicular destruction. Clinical and dermoscopic findings may include vertex-centered thinning, perifollicular erythema or scale, peripilar white-gray halos, and progressive loss of follicular openings [3,4]. Representative features are shown in Figure 1. Recognition of these findings may facilitate earlier diagnosis, patient education, and intervention before extensive scarring develops.

**Figure 1:**
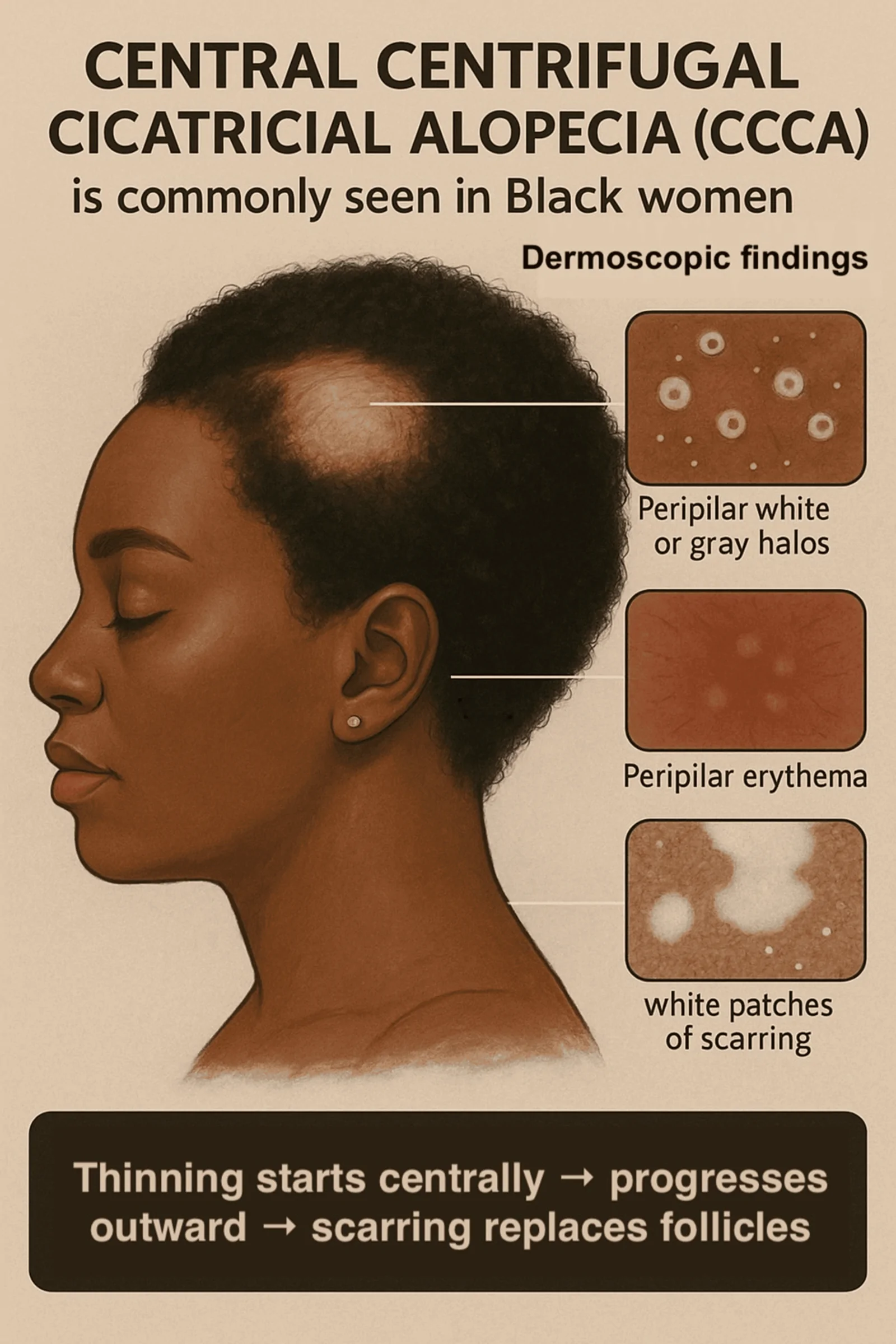
Clinical and dermoscopic features of CCCA in Black women Image credit: The authors, using Adobe Acrobat (Adobe Inc., San Jose, CA, USA) and Microsoft Paint (Microsoft Corporation, Redmond, WA, USA). CCCA: central centrifugal cicatricial alopecia

Historically referred to as “hot-comb alopecia” or “follicular degeneration syndrome,” CCCA was previously attributed primarily to cultural hairstyling practices. Current evidence instead supports a multifactorial pathogenesis involving genetic susceptibility, immune dysregulation, fibroproliferative activity, metabolic abnormalities, and possible environmental contributors [4-6]. Genetic studies have identified peptidyl arginine deiminase 3 (PADI3) variants in a subset of affected individuals, while molecular investigations have reported altered fibroproliferative and immune-related gene-expression patterns [4-8]. The clinical significance of these findings and their potential relationship to disease severity, prognosis, and treatment response remain under investigation.

Beyond permanent hair loss, CCCA may substantially affect psychosocial well-being and quality of life. Affected individuals may experience self-consciousness, sadness, and distress related to visible scalp involvement and progressive hair loss [9,10]. However, available evidence regarding formal associations with anxiety and depressive disorders remains limited and inconsistent [1,11]. Clinical management should therefore consider both the physical and psychosocial effects of the disease.

Recent reviews have examined the epidemiology, risk factors, clinical presentation, and evolving pathogenesis of CCCA, while expert consensus recommendations have provided guidance regarding treatment [1-3]. This review builds on that literature by integrating clinical differentiation from common alopecia mimickers with genetic, transcriptomic, proteomic, fibroinflammatory, and metabolic findings published between 2015 and 2025. It also distinguishes established management approaches from emerging or investigational therapies and identifies areas in which the evidence remains limited or inconclusive. Accordingly, this review aims to describe the diagnostic challenges associated with CCCA, including its differentiation from other alopecias; synthesize recent genetic and molecular findings related to disease pathogenesis; and critically evaluate current management strategies and potential therapeutic directions.

## Review

Methods

A structured literature search of PubMed, Scopus, and Web of Science was conducted in November 2025 for articles published between January 1, 2015, and November 30, 2025. Search terms included combinations of “central centrifugal cicatricial alopecia,” “CCCA,” “scarring alopecia,” “cicatricial alopecia,” “Black women hair loss,” and terms related to diagnosis, dermoscopy, histopathology, pathogenesis, genetics, comorbidities, and treatment. The reference lists of eligible studies, recent reviews, and consensus publications were also examined. Seminal articles published before 2015 were included when necessary to provide historical, diagnostic, or mechanistic context but were not counted among the primary studies.

The search identified approximately 365 records. After duplicate and clearly irrelevant records were removed, titles and abstracts were screened according to the objectives of the review. Articles potentially reporting original CCCA-specific data on epidemiology, clinical presentation, diagnosis, dermoscopy, histopathology, molecular or genetic mechanisms, comorbidities, or treatment underwent full-text assessment. Full texts were also examined when eligibility could not be determined from the title and abstract.

Primary studies were eligible if they reported original human data; included a clearly identifiable population with clinically or histopathologically diagnosed CCCA; presented CCCA-specific findings separately from those concerning other alopecias; addressed at least one prespecified review domain; provided sufficient methodological detail to interpret the population, exposure or intervention, and outcomes; and were available as full-text publications in English. Eligible designs included cohort, case-control, cross-sectional, molecular, genetic, clinicopathologic, and clinically informative case-series studies. Studies involving Black women were particularly relevant because CCCA disproportionately affects this population, although eligibility was not restricted by sex, race, or ethnicity.

Studies were excluded if they duplicated a previously reported population; combined multiple alopecia diagnoses without presenting separate CCCA findings; inadequately defined the diagnosis; lacked sufficient methodological detail; reported only nonhuman findings without direct clinical relevance; or were available only as conference abstracts. Studies of other hair disorders were excluded unless they included a distinct CCCA subgroup or contributed specifically to the differential diagnosis. When publications described overlapping cohorts, the report containing the most complete and relevant CCCA-specific data was prioritized. Following full-text review against these eligibility criteria, 15 studies met the requirements for final inclusion in this manuscript. Selection was based on fulfillment of the eligibility criteria, irrespective of the direction or statistical significance of the findings.

Review articles, consensus recommendations, and case reports were used as supplemental evidence but were not included among the 15 primary studies. Reviews provided context and assisted in identifying additional publications, consensus statements described areas of expert agreement, and case reports informed the discussion of uncommon presentations or emerging therapies for which stronger evidence was unavailable.

No formal risk-of-bias assessment or standardized critical-appraisal instrument, such as the Joanna Briggs Institute checklists, was applied. Instead, methodological limitations, including retrospective design, small sample size, single-center recruitment, reliance on diagnostic coding, inadequate controls, selection bias, and insufficient adjustment for confounding, were considered qualitatively. Greater interpretive weight was assigned to controlled studies, larger cohorts, clearly defined diagnostic criteria, and appropriately adjusted analyses. The absence of a formal quality appraisal is acknowledged as a limitation of this narrative review.

Data were extracted on study design, population characteristics, sample size, diagnostic criteria, exposures or interventions, outcomes, principal findings, and reported limitations. Results were organized into epidemiology and risk factors, clinical presentation and differential diagnosis, dermoscopy and histopathology, genetic and molecular pathogenesis, comorbidities, and management. Because of substantial heterogeneity in study designs, populations, exposures, and outcomes, meta-analysis was not considered appropriate. Findings were synthesized descriptively, and evidence from case reports and small case series was considered hypothesis-generating rather than confirmatory.

Results

Epidemiology and Clinical Presentation

CCCA mainly occurs in women of African descent, most commonly between the third and fifth decades of life. This condition manifests infrequently in Black men but remains extremely uncommon across other racial demographics without clear behavioral patterns. The onset of CCCA typically involves indiscernible vertex thinning, which gradually expands in a centrifugal manner. Heightened fragility or breakage at the crown may precede overt alopecia and could be an early clinical indicator [12].

In the early inflammatory stages, patients may experience symptoms such as itching, burning, or tenderness, often accompanied by erythema, perifollicular scale, or small pustules [13]. As the disease progresses, ongoing inflammation and fibrosis lead to more widespread alopecia. Suchonwanit (2016) found that longer disease duration was significantly associated with increased severity [14]. Similarly, Roche and colleagues (2022) reported an association between advancing age and histologic progression in CCCA [15]. In the absence of intervention, disease progression is typically expected to continue, and the concurrent inflammatory and scarring phases are believed to contribute to the ongoing destruction of hair follicles [14,16].

Involvement of the central scalp area is typical, but atypical presentations, including patchy, lateral, and even bitemporal distributions, have also been described. These atypical patterns can mimic other forms of alopecia and contribute to a delayed diagnosis [12,17]. Recognition of these variants is crucial to avoid misclassification and ensure that a biopsy is performed promptly.

Diagnostic Challenges and Differential Diagnosis

Diagnosis of CCCA can be challenging, as early disease may mimic more common non-scarring alopecias. In the early, diffuse thinning phase, CCCA may mimic female pattern hair loss (androgenetic alopecia), which also causes diffuse thinning of the vertex [17]. However, while androgenetic alopecia is characterized by dermoscopic findings of miniaturized hairs of varying diameters with preserved follicular openings, CCCA demonstrates loss of openings and scarring [1,3]. Alopecia areata may also present similarly in appearance, manifesting as a patch of crown hair loss with exclamation point hairs and intact ostia; indeed, one case was misdiagnosed as CCCA [18-20].

CCCA must also be differentiated from other scarring alopecias. Lichen planopilaris (LPP) causes patchy hair loss with perifollicular scale. LPP is most often found at the frontal and parietal scalp but may involve the crown. Histopathology of LPP and CCCA has significant overlap, with both lesions commonly showing lichenoid lymphocytic infiltrates. In this case, clinicopathologic correlation is required. Frontal fibrosing alopecia (FFA), a variant of LPP, is limited to the hairline and eyebrows and, therefore, is rarely confused with CCCA. Traction alopecia, secondary to chronic tension from braids/weaves, most commonly affects the frontal scalp where most traction is imparted [21]. The characteristic clinical patterns of CCCA and its common mimickers are illustrated in Figure 2. Their distinguishing clinical, dermoscopic, and histopathologic features are summarized separately in Table 1.

**Figure 2:**
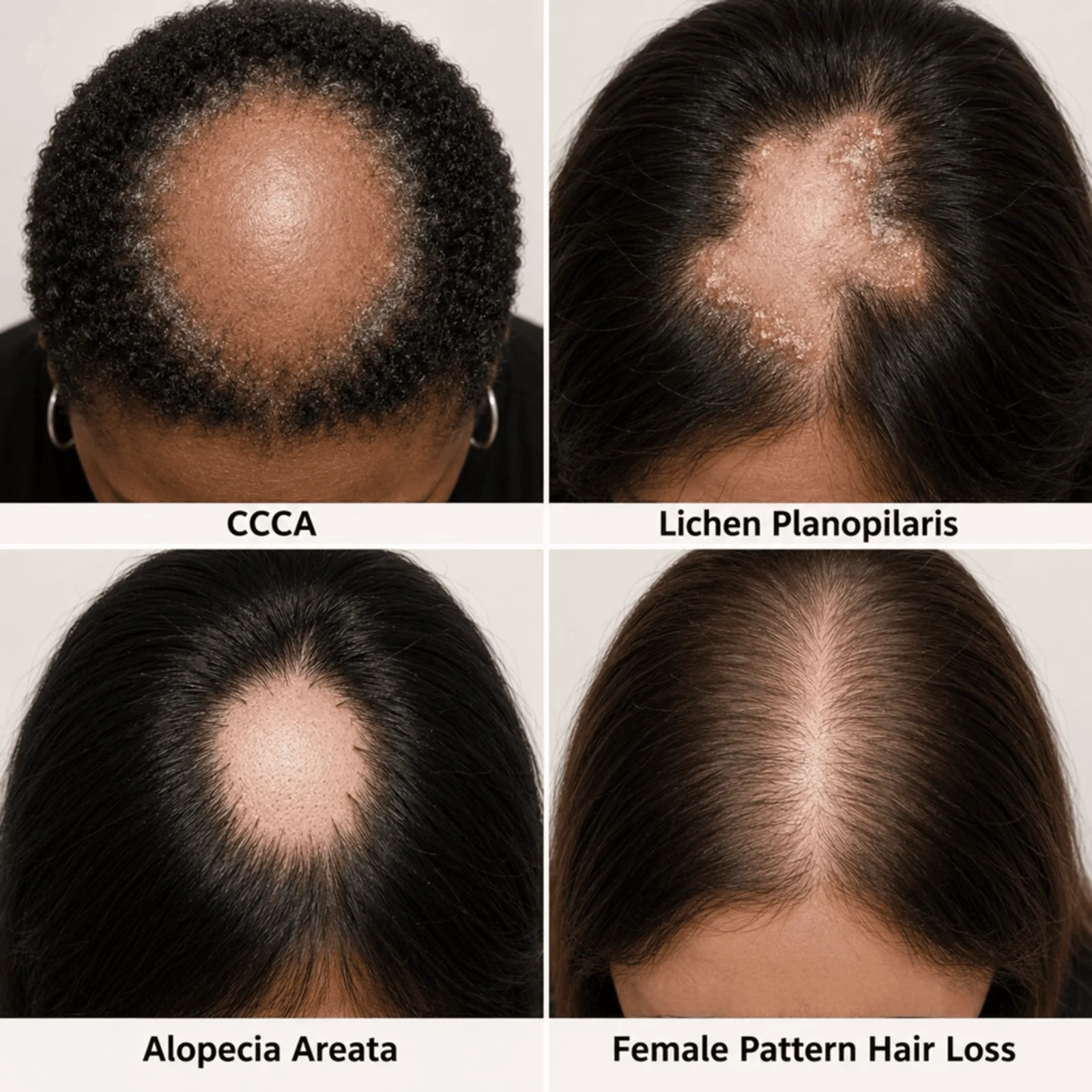
Clinical patterns of central centrifugal cicatricial alopecia (CCCA) and common mimickers

**Table 1:** Comparative clinical and histologic features of common alopecias CCCA: central centrifugal cicatricial alopecia; FFA: frontal fibrosing alopecia

Feature	CCCA	Lichen Planopilaris	Alopecia Areata	Female Pattern Hair Loss
Demographics	Black women, age 30–60 [1,2]	Women > men, mid-age, all ethnicities [18]	Any sex, younger onset [19]	Women post-puberty, often postmenopause [20]
Pattern	Vertex → outward circle [1]	Scattered vertex/frontal; FFA = frontotemporal [21]	Localized patches ± ophiasis [19]	Diffuse crown thinning; frontal spared [20]
Scarring	Yes, lost ostia [16]	Yes, perifollicular scarring [18]	No, ostia intact [19]	No, miniaturized follicles [20]
Symptoms	Itch, burn, tender [3,5]	Itch/burn common [18]	Usually none [19]	None or mild sensitivity [20]
Clues	Central shiny bald patch ± scale/pustules [1,5]	Perifollicular scale/erythema; FFA recession; lonely hair [21]	Exclamation hairs; yellow/black dots; nail pitting [19]	Hair shaft variability; diffuse crown loss [20]
Histology	Lymphocytic peri-infundibular infiltrate; concentric fibrosis [4,22]	Lichenoid infiltrate; perifollicular fibrosis; dropout [18]	Peribulbar lymphocytes; no fibrosis [19]	Follicle miniaturization; ↑ telogen; no inflammation [20]

Table 1 demonstrates that loss of follicular openings is a major feature that separates CCCA-type scarring alopecia from the non-scarring type. In more subtle cases, a scalp biopsy is mandatory, and dermoscopy may help direct the biopsy site by recognizing the perifollicular scale and the active border. Histology will reveal lymphocytic inflammation at the isthmus/infundibulum in early disease and replacement of the follicle by a fibrous tract in more chronic cases. Premature desquamation of the inner root sheath has been recently postulated as a more specific marker in the early phase of the disease [23]. Since end-stage fibrosis is a common finding in advanced CCCA, as well as in LPP, and CCCA may coexist with traction or androgenetic alopecia, an accurate diagnosis can only be made by taking into consideration the history, dermoscopy, and expert biopsy review (with repeat sampling when necessary) [1].

Molecular Insights Into Pathogenesis

CCCA is increasingly thought to be a complex, multifactorial disorder involving genetic predisposition, immune dysregulation, and aberrant fibrosis pathways as the driving forces of the disease, rather than the result of traction or hairstyling. Environmental conditions may trigger or exacerbate the disease, but biological abnormalities are the primary contributing factor, a notion supported by research conducted over the past few decades.

Genetic evidence suggests that inherited factors may contribute to CCCA, as the condition is more commonly observed among biologically related family members [24]. Malki (2019) performed a gene analysis and discovered PADI3 variants associated with hair shaft abnormalities [25]. A separate study reported ~25% of patients carried autosomal-dominant PADI3 mutations [6]. Other studies have found upregulation of genes that promote fibroproliferation and incomplete penetrance, with other fibrotic disorders, suggesting that this is a key molecular component of the disorder [2,4]. CCCA has also been linked to uterine fibroids, a common fibroproliferative disorder among women of African descent [4]. Population-genetic analyses have identified evidence consistent with selection favoring fibroproliferative disease risk alleles in populations of African ancestry, although these findings are not specific to CCCA [26]. Two separate studies have shown that these patients face an increased risk of fibroid development [27,28], while another study contradicted these findings [29]. In general, CCCA is polygenic, involving genes related to both follicular structure and fibrosis.

Active CCCA lesions exhibit perifollicular T-cell infiltrates, which demonstrate a chronic inflammatory response [22]. The histologic feature aligns with lymphocytic alopecias, such as alopecia areata and LPP, which have documented autoimmune mechanisms [18]. Further support for autoimmune processes in CCCA has been recently elucidated. Ong (2025) found ANA positivity in 11.8% of patients with CCCA, compared to 2% of controls, and noted an increased prevalence of lupus. Most patients tested in this study did not meet complete connective tissue disease criteria, which suggests CCCA might be an organ-targeted autoimmune condition or share systemic autoimmunity risk factors [30,31]. Taken together, these studies have further clarified CCCA as a potential systemic marker for immune dysfunction in addition to a localized follicular disorder.

The progressive fibrosis that replaces destroyed hair follicles is a hallmark of CCCA and results from continuous inflammation. Molecular investigations have focused on the role of peroxisome proliferator-activated receptor gamma (PPAR-γ), whose loss of function causes scarring alopecia in mice and is downregulated in human lesions [1,10]. A recent transcriptomic meta-analysis found gene-expression patterns associated with fibrosis across several primary lymphocytic scarring alopecias. In CCCA samples, the analysis also identified reduced expression of genes involved in peroxisomal activity, lipid metabolism, mitochondrial function, and epithelial maintenance. These findings suggest, but do not establish, that altered immune and metabolic pathways may contribute to progressive follicular damage in CCCA [32]. In some cases, PPAR-γ activity may be initially low, leading to an impaired healing process and fibrosis [33]. In its absence, mediators of fibrosis, such as transforming growth factor β (TGF-β), become predominant and lead to collagen deposition. PPAR-γ agonists have been partially beneficial in the treatment of LPP, and thus may be relevant to CCCA [34].

Excisional scalp biopsies from CCCA reveal enhanced fibrogenic cytokines, with TGF-β serving as a principal mediator of fibrosis and the nexus of the transition from inflammatory activity to scarring [6]. Repetitive insult and/or immune-mediated attack contribute to aberrant wound healing processes, where fibrosis overrides regeneration (fibroplasia), a process modulated by genetic determinants. IL-13, a key player in pulmonary fibrosis, is currently being explored as a potential antifibrotic therapeutic target in scarring alopecia [1]. Persistent environmental factors (traction, relaxers) induce follicular injury. In contrast, genetic predisposition (mutations in PADI3 and decreased PPAR-γ) and autoimmune features (ANA positivity and lupus) heighten the inflammatory response, culminating in irreversible fibrosis through TGF-β signaling pathways [31,35]. These parallel pathways account for why only some exposed women develop CCCA and justify antifibrotic therapy when scarring predominates [34].

Risk Factors and Comorbidities

Genetic and immunologic mechanisms contribute to the pathogenesis of CCCA, while environmental and systemic factors may influence disease onset and clinical expression [10,25]. Hairstyling practices are the most extensively studied potentially modifiable factors associated with CCCA [1]. Traction-producing hairstyles, including tight braids and weaves, may expose susceptible follicles to repeated mechanical stress; however, findings across studies are inconsistent, and causality has not been established [1,21]. Examples of traction-producing hairstyles and their associated patterns of marginal hair loss are illustrated in Figure 3. Evidence regarding chemical relaxers is similarly inconclusive, with some studies reporting an association and others finding no statistically significant relationship [1,36]. Chemical burns, excessive heat, and improper product application may directly injure the scalp and hair follicles, although their specific contribution to CCCA remains uncertain [2,13]. Hot-comb use was historically implicated in “hot-comb alopecia”; however, current evidence indicates that CCCA is multifactorial and cannot be attributed to heat exposure or any single hairstyling practice [2,5].

**Figure 3:**
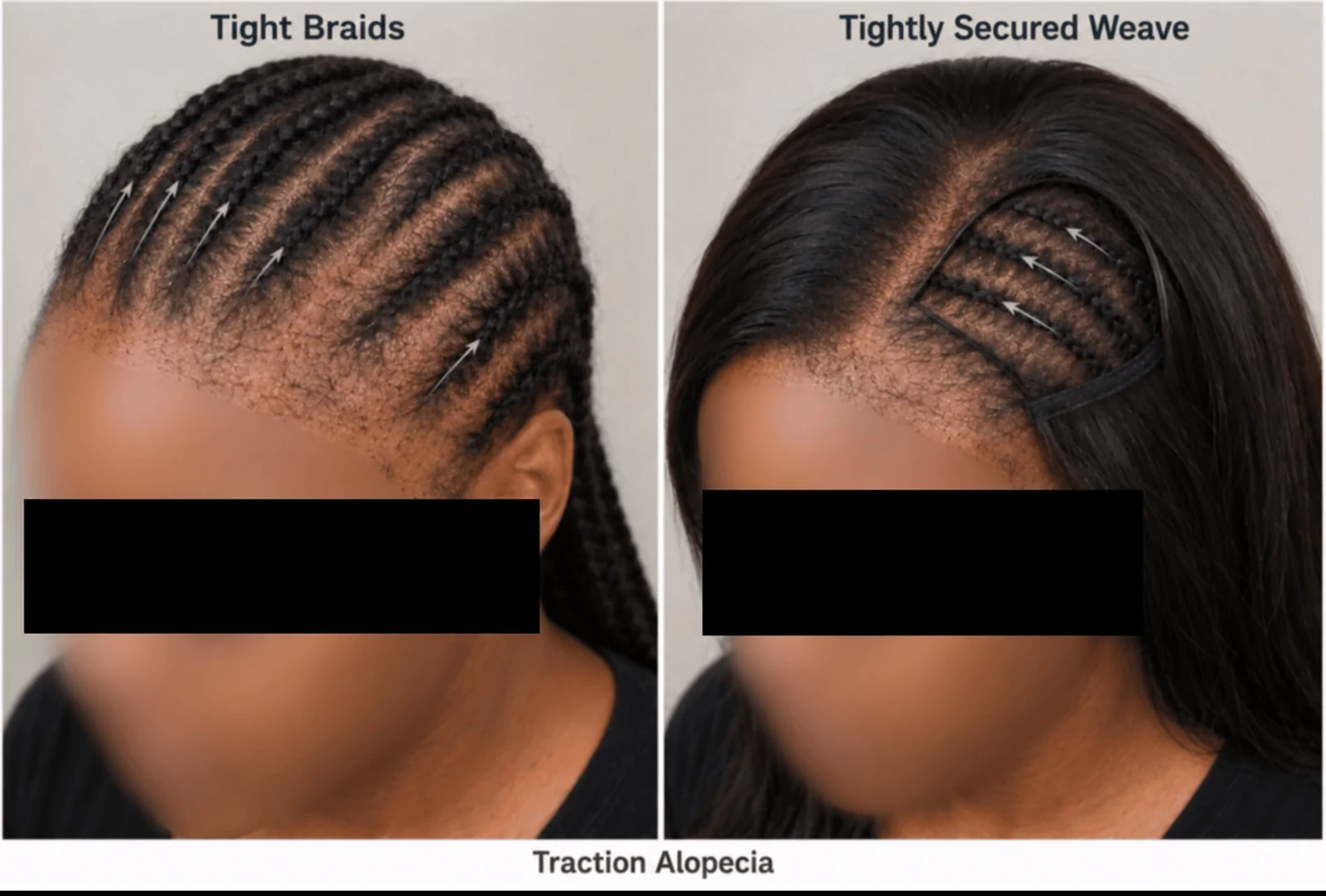
Traction alopecia associated with tight braids and weaves

Metabolic comorbidities represent another area of interest. Proposed relationships among insulin resistance, altered PPAR-γ signaling, and fibrosis provide a biologically plausible framework for further investigation but remain unconfirmed [33]. Observational studies have examined potential associations between CCCA and metabolic conditions, including type 2 diabetes, obesity, and dyslipidemia; however, the reported findings have been inconsistent [35].

Reproductive and endocrine factors have also been investigated. One retrospective case-control study of 74 women with CCCA found greater odds of previous pregnancy and chemical-relaxer use compared with age-, sex-, and race-matched controls with other hair-loss disorders [36]. However, the observational design precludes causal inference, and the pregnancy association has not been independently replicated. Available evidence also does not establish a consistent association between CCCA and androgen excess or polycystic ovary syndrome [14]. Additional studies are needed to determine whether reproductive or endocrine factors influence CCCA susceptibility or progression.

The Black Women’s Health Study provides a large prospective cohort through which metabolic and dermatologic outcomes among Black women may be examined [37]. An analysis from this cohort found that type 2 diabetes was associated with increased odds of severe central-scalp hair loss; however, this phenotype was not specific to CCCA [38]. Other observational studies have reported associations between type 2 diabetes and CCCA, although the overall evidence remains inconclusive and does not establish causality [1]. Findings regarding obesity and dyslipidemia have also varied across studies [35,39].

Micronutrient status, particularly vitamin D deficiency, has received increasing attention. A retrospective study reported vitamin D deficiency or insufficiency in 92.6% of Black patients with CCCA and found greater odds of deficiency or insufficiency compared with estimates for the general Black population [40]. However, the study included only 27 eligible patients and did not use an internal control group, limiting the strength and generalizability of its findings. Vitamin D and vitamin D receptor signaling contribute to hair-follicle integrity, follicular cycling, keratinocyte differentiation, and cutaneous immune regulation [41].

A subsequent multisite study of 240 patients with primary scarring alopecia identified vitamin D deficiency in 56.6% of the overall cohort and 68.4% of patients with CCCA. Patients with CCCA also had greater odds of vitamin D deficiency than patients with other primary scarring alopecias [28]. A systematic review and meta-analysis similarly reported vitamin D deficiency among patients with primary scarring alopecia, although limited disease-specific data prevented definitive conclusions regarding CCCA [42]. These observational findings demonstrate an association but do not establish that vitamin D deficiency contributes to CCCA development or progression. Greater skin pigmentation may reduce cutaneous vitamin D synthesis because melanin absorbs ultraviolet B radiation; however, vitamin D status is also influenced by geographic, environmental, behavioral, dietary, and genetic factors [43]. Vitamin D has not been established as a CCCA-specific biomarker or disease-modifying therapeutic target. Accordingly, evaluation and treatment of vitamin D deficiency should follow established clinical indications rather than being presented as CCCA-specific management [28,40-43].

Management and Treatment Strategies

Successful CCCA management aims to eliminate follicular inflammation, prevent further damage, and, when possible, restore affected areas. Once hair follicles are irreversibly replaced by fibrotic tissue, regrowth is no longer possible. Accordingly, treatment is aimed at arresting disease progression, preserving remaining viable follicles, and optimizing cosmetic outcomes. Management should be individualized according to disease activity, classified as active or inactive, as well as relevant patient-specific clinical factors.

Clinicians should begin by obtaining a detailed hair loss history, including styling techniques and a family history of other scarring alopecias [28]. Scalp biopsy is recommended when suspicion remains high to confirm diagnosis and exclude mimics like LPP [18]. In untreated CCCA, first-line therapy often involves potent topical corticosteroids (e.g., clobetasol propionate 0.05% solution or foam) and intralesional triamcinolone injections every four to eight weeks. These agents can suppress inflammation, relieve pruritus, and may stabilize or modestly regrow hair when applied early in the disease course [1,5]. The adjunctive use of antimicrobial shampoos containing zinc pyrithione or ketoconazole can help improve the scalp microenvironment and manage coexistent seborrheic dermatitis [1]. Patients should be counseled to avoid tight hairstyles, chemical relaxers, and excessive heat, as these may perpetuate follicular injury [1,21].

Systemic therapy is generally considered for active CCCA that does not respond adequately to first-line treatment. Tetracyclines, including doxycycline and minocycline, may be used for their anti-inflammatory and matrix metalloproteinase-inhibitory effects; however, the supporting evidence is stronger for LPP than for CCCA [18,23]. In severe or treatment-refractory cases with ongoing inflammation, systemic immunosuppressive agents such as methotrexate or cyclosporine may be considered, although evidence supporting their use in CCCA remains limited [13].

Agents with antifibrotic or reparative potential represent an emerging approach to CCCA treatment. Pioglitazone, a PPAR-γ agonist approved for diabetes, has been associated with hair regrowth in case reports involving several forms of cicatricial alopecia [44,45]. Mechanistic evidence further supports the importance of PPAR-γ signaling in the pathogenesis of CCCA [35]. Similarly, Janus kinase (JAK) inhibitors, such as baricitinib, have produced hair regrowth in a case series of patients with refractory CCCA [46]. However, their off-label use requires careful patient selection and monitoring because of the potential risks of serious infections and thrombosis.

Metformin, a first-line medication for type 2 diabetes, has been repurposed for its antifibrotic effects, which are mediated in part through activation of AMP-activated protein kinase (AMPK). In a pilot study, low-dose metformin resulted in symptomatic improvement and reduced expression of profibrotic genes [9]. Topical metformin was also associated with hair regrowth in two patients with refractory CCCA [47]. More recently, a case report and literature review described clinical improvement with topical 10% metformin cream, further supporting its potential role as an adjunctive antifibrotic therapy in CCCA [48].

Platelet-rich plasma (PRP) therapy, which involves collecting and centrifuging autologous blood to concentrate platelets before reinjection into the scalp, has also demonstrated encouraging results. In one study, PRP improved disease severity, and 75% of treated patients experienced increased hair density after six months [49]. Table 2 summarizes these treatment modalities, their proposed mechanisms of action, and the corresponding levels of supporting evidence.

**Table 2:** Treatment modalities for central centrifugal cicatricial alopecia (mechanisms and evidence) CCCA: central centrifugal cicatricial alopecia; MMP: matrix metalloproteinase; LPP: lichen planopilaris; JAK: Janus kinase; AMPK: AMP-activated protein kinase; PRP: platelet-rich plasma; QoL: quality of life

Therapy	Mechanism/Rationale	Evidence/Notes
Topical corticosteroids [1,5]	Anti-inflammatory	First-line; slows progression; clobetasol is common; monitor for atrophy
Intralesional steroids [1]	Local anti-inflammatory	Triamcinolone reduces itch/inflammation
Topical calcineurin inhibitors [18]	T-cell suppression, steroid-sparing	Second-line/maintenance; limited CCCA data
Tetracyclines [18,23]	Anti-inflammatory, MMP inhibition	Off-label; useful in inflammatory phase
Hydroxychloroquine [18]	Immunomodulator	Anecdotal; LPP evidence; eye monitoring needed
Oral minoxidil [1]	Promotes growth, ↑blood flow	Emerging; thickens miniaturized hairs, no scar reversal
PPAR-γ agonist (pioglitazone) [32]	Restores signaling, ↓fibrosis	Case reports; experimental
JAK inhibitors [48]	Block JAK-STAT inflammation	Single case (baricitinib); costly, immunosuppressive
Metformin [47]	Anti-fibrotic, AMPK activation	Pilot: ↓ fibrosis markers; topical under study
PRP [48]	Growth factor delivery	Early: ~75% improved density; requires repeats
Hair transplant [1,4]	Follicular transfer	Consider if inactive ≥12 mo; variable success
Wigs/camouflage [1]	Cosmetic	Improves QoL; complements medical care

Surgical and cosmetic management are reserved for patients with inactive disease (≥6-12 months). Follicular unit transplantation can offer cosmetic improvement but is technically challenging due to compromised vasculature in a scarred scalp; therefore, it is essential to confirm disease quiescence to prevent graft loss [42,50]. Wigs and camouflage play a crucial role in supportive care, optimizing patient quality of life [1]. Figure 4 provides a stepwise algorithm to guide management at various stages of the disease.

**Figure 4:**
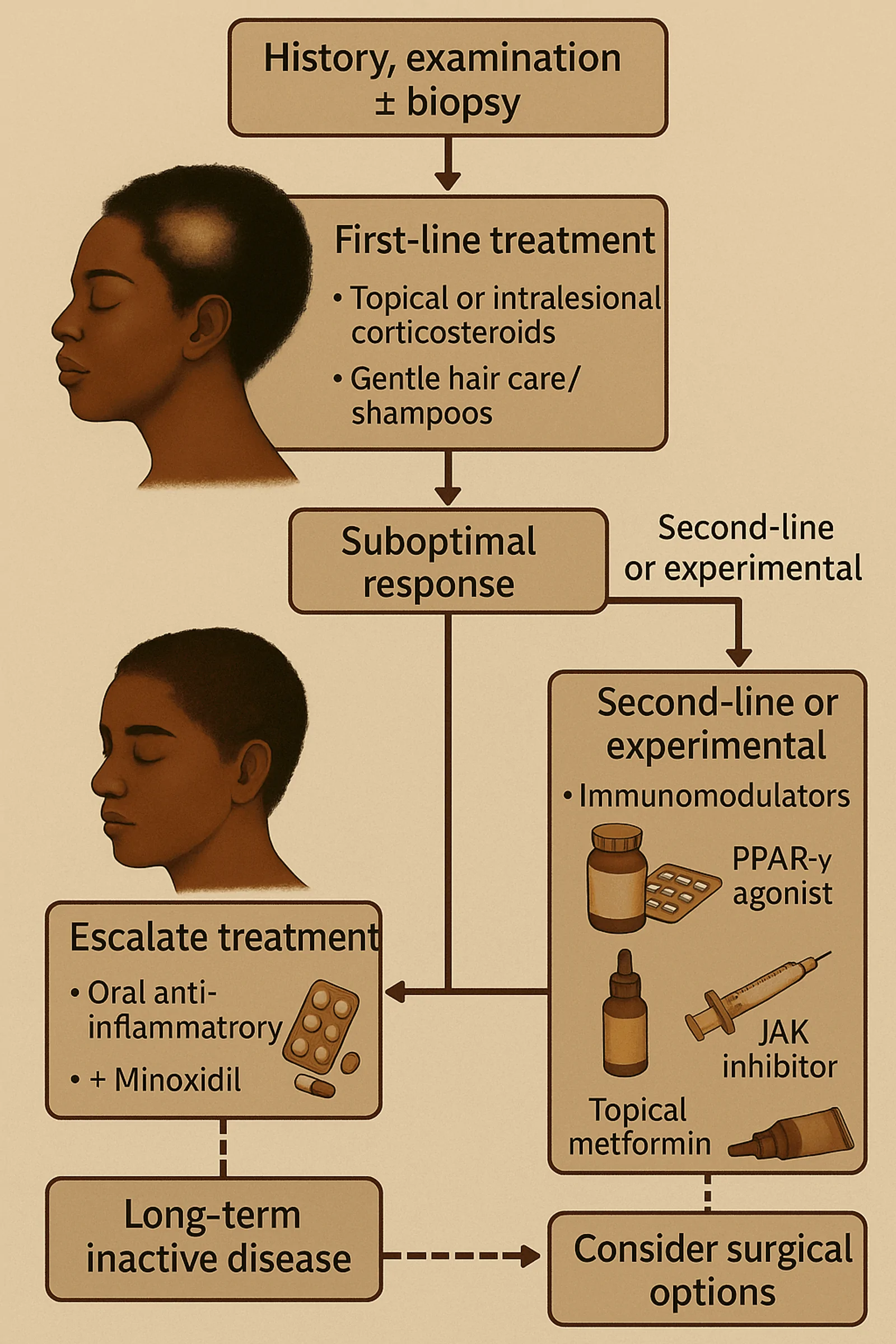
Proposed treatment algorithm for central centrifugal cicatricial alopecia Image credit: The authors, using Adobe Acrobat (Adobe Inc., San Jose, CA, USA) and Microsoft Paint (Microsoft Corporation, Redmond, WA, USA). PPAR-γ: peroxisome proliferator-activated receptor gamma; JAK: Janus kinase

Providers may also employ combination approaches (e.g., topical steroids with tetracyclines, followed by oral minoxidil) and then transition to maintenance with tacrolimus. Co-management of other comorbid conditions, such as diabetes or lupus, may optimize systemic and scalp disease. In the absence of extensive trials, most treatment recommendations are derived from case series, consensus, and shared decision-making [23]. Re-evaluation and regular follow-up should be scheduled with close clinical monitoring. However, if no response is observed after approximately six months, it may be reasonable to switch therapy.

Overall, CCCA appears to result from a mix of genetic, immune, and environmental factors that cause inflammation and scarring of the hair follicles. It most often affects women of African descent and can present in different patterns, which makes diagnosis challenging. Treatment focuses on reducing inflammation, halting disease progression, and preventing further hair loss, with new therapies showing early promise. Continued research is needed to better understand risk factors, improve diagnosis, and develop more effective treatment options.

Discussion

In Black women, CCCA represents a nexus of two seemingly divergent concepts: cultural hair practices and genetic or immune predisposition. Though previously ascribed largely to hairstyling, our increasing understanding of its etiology has revealed CCCA to be a multifactorial disorder. Certain hairstyles may act as precipitants or potentiators of the disease; however, PADI3 mutations and fibroproliferative gene expression place CCCA at the confluence of other scarring alopecias and fibrotic disorders of different organ systems [46]. Delayed diagnosis remains a significant barrier, as early disease is often mistaken for styling-related damage or androgenetic alopecia. Many patients present only after extensive fibrosis, when therapeutic options are limited. Greater awareness among clinicians, along with dermoscopy training, could enable earlier recognition of hallmark signs such as perifollicular scale and absent ostia [10]. Antifibrotic and immunomodulatory therapies have been studied based on an understanding of the pathogenesis, including pirfenidone, IL-13 blockers, antimalarials, and JAK inhibitors. However, most current treatment modalities are extrapolated from other scarring alopecias [1,6].

Whether asymptomatic or mild CCCA should be treated remains debated. In clinical practice, treatment decisions should be individualized by balancing the risk of irreversible hair loss against the potential adverse effects of therapy. The reported association between CCCA and metabolic syndrome has prompted greater interest in the role of metabolic dysfunction in disease pathogenesis. Acanthosis nigricans is relevant because it is a clinical marker of insulin resistance, a central feature of metabolic syndrome; however, it is not considered a direct manifestation or established cause of CCCA [33]. Patients with CCCA may therefore benefit from evaluation for metabolic and other associated comorbidities, with multidisciplinary care when indicated. Bao et al. (2025) further proposed that metabolic dysregulation may contribute to lymphocytic cicatricial alopecias, although additional research is needed to determine whether this relationship is causal [9].

In summary, the complexities of CCCA span a range of genetic, immune, and external triggers that are modified by additional systemic comorbidities. Education and awareness of the disease among clinicians, as well as increased vigilance and earlier recognition, are some of the most critical solutions. Precision-based targeted therapies, as they become available, should be used where appropriate and could slow disease progression; however, they will need to be approached with an understanding of the disease's unique and complicated care continuum.

## Conclusions

CCCA is a chronic, multifactorial scarring alopecia that predominantly affects Black women and may cause permanent hair loss. Although historically attributed primarily to hairstyling practices, current evidence suggests a complex pathogenesis involving genetic susceptibility, immune dysfunction, altered wound healing, and potential environmental contributors. Early recognition is essential to limit irreversible follicular damage. Dermoscopy can support diagnosis, while scalp biopsy may be used when clinical findings are uncertain or histopathologic confirmation is needed. Established management focuses on patient education, gentle hair-care practices, and anti-inflammatory treatment to control symptoms and slow disease progression. Minoxidil may be used as an adjunct to support hair growth in follicles that remain viable. Evidence for PRP remains limited, and its role in routine management has not been established. Molecularly targeted treatments, including pioglitazone and JAK inhibitors, remain investigational and should not be considered standard therapy without stronger clinical evidence. Hair transplantation may be considered in carefully selected patients with stable, inactive disease. Further research is needed to clarify disease mechanisms, evaluate emerging therapies, and develop more individualized approaches that improve outcomes and access to care.
